# Evidence of neurocognitive and resting state functional connectivity differences in carriers of *NRXN1* deletions

**DOI:** 10.1186/s11689-025-09625-5

**Published:** 2025-10-15

**Authors:** Jacqueline Fitzgerald, Ciara J. Molloy, Thomas Dinneen, Niamh E. Feerick, Matthew O’Sullivan, Richard O’Conaill, Maryam Al-Shehhi, Richard Reilly, Sally Ann Lynch, Eleisa A. Heron, Clare Kelly, Sanbing Shen, Louise Gallagher

**Affiliations:** 1 Department of Psychiatry, School of Medicine, Trinity College Dublin, Dublin, Ireland; 2 Trinity Centre for Biomedical Engineering, Trinity College Dublin, Dublin, Ireland; 3 Children’s Health Ireland, Crumlin, Dublin 12, Ireland; 4 Sheikh Khalifa Medical City, Abu Dhabi, UAE; 5 Children’s Health Ireland, Temple St., Dublin, Ireland; 6 Academic Center On Rare Diseases, School of Medicine and Medical Science, University College Dublin, Dublin, Ireland; 7 School of Psychology, Trinity College, Dublin, Ireland; 8 Trinity College Institute of Neuroscience, Trinity College, Dublin, Ireland; 9 Regenerative Medicine Institute, School of Medicine, (NUI) Galway, Galway, Ireland; 10 The Hospital for Sick Children, Toronto, Canada; 11 The Peter Gilgan Centre for Research and Learning, SickKids Research Institute, Toronto, Canada; 12 Child and Youth Mental Health Collaborative, The Hospital for Sick Children, The Centre for Addiction and Mental Health, Toronto, Canada; 13 Department of Psychiatry, Temerty Faculty of Medicine, University of Toronto, Toronto, Canada

**Keywords:** Neuroimaging, Magnetic resonance imaging, Cognition, Copy number variant, NRXN1 deletion

## Abstract

**Background:**

*NRXN1* deletion (*NRXN1* del) is a rare copy number variant associated with several neurodevelopmental, neuropsychiatric, and cognitive outcomes. The *NRXN1* gene encodes for a pre-synaptic cell adhesion molecule that is important for synapse formation, regulation and neurotransmission. We used a gene-first approach to investigate neurocognitive and brain phenotypes in *NRXN1* d*e*l carriers.

**Methods:**

Forty-two participants (21 *NRXN1* del carriers and 21 neurotypical age and sex-matched comparisons) completed IQ assessments, and a neurocognitive battery, including, executive function, attention, and social cognition tasks. Magnetic resonance imaging (MRI) data, including T1-weighted anatomical scans, resting state functional MRI and diffusion tensor imaging, were acquired in 36 participants (17 *NRXN1* del carriers and 19 comparisons).

**Results:**

*NRXN1* del carriers had lower mean IQ and poorer spatial working memory performance compared to comparisons (*p* ≤ 0.05). Neuroimaging results revealed group differences in visual and ventral attention resting state networks (*p* < 0.05). Network-based statistical analysis showed a significant effect of group status for 28/115 connections, with poorer segregation between visual and default networks in *NRXN1* del carriers relative to comparisons. No differences in brain structural volume or cortical thickness, or diffusion measures of white matter structural architecture were observed between groups.

**Conclusions:**

This exploratory study provides evidence for neurocognitive impacts and brain functional differences related to underlying synaptic mechanisms. Brain functional differences in *NRXN1* del carriers may support altered excitation/inhibition dynamics within the brain. Gene-first approaches may establish brain-based translational markers to identify neurobiologically informed subgroups within neurodevelopmental and neuropsychiatric conditions, and ultimately transdiagnostic therapeutic strategies.

**Supplementary Information:**

The online version contains supplementary material available at 10.1186/s11689-025-09625-5.

## Background

*NRXN1* deletion (*NRXN1* del*)* is one of several rare copy number variants (CNV) associated with increased likelihood for neuropsychiatric and neurodevelopmental conditions (ND-CNV) including autism, intellectual disability, and schizophrenia [15, 30, 37, 38]. Cognitive differences are also reported in both clinical and general population ND-CNV cohort studies [33, 34, 47]. *NRXN1* del is associated with a wide range of cognitive, language, psychiatric and neurological outcomes in both clinical and population-based studies, and occurs at a reported prevalence of 2.55/1000 individuals in the general population [48].

The *NRXN1* gene, located at 2p16.3, spans 24 exons over 1.1 Mb, [57, 66]. As one of the largest known human genes, it is susceptible to structural variation [23]. *NRXN1* dels may arise de novo or be inherited, and are enriched in clinical neurodevelopmental cohorts compared with the general population [16, 22, 37, 55]. Most clinically relevant *NRXN1* dels are exonic, particularly those affecting early exons (e.g. 1–5), which are enriched in neurodevelopmental cohort. However, some intronic deletions may also have functional consequences, especially those disrupting splice sites or regulatory regions [18].

*NRXN1* encodes for a type I membrane presynaptic cell-adhesion molecule known to bind to post-synaptic ligands including neuroligins (NLGNs), leucine-rich repeat transmembrane proteins (LRRTMs) and cerebellins [26, 65]. *NRXN1* encodes two major isoforms; a long alpha (α) arm and a short beta (β) arm [45, 64, 66, 70]. The majority of *NRXN1* CNV deletions reported in clinical and population cohorts affect *NRXN1*α [43]. *NRXN*s are essential for calcium dependent excitatory and inhibitory neurotransmission [21, 45], as well as promoting synapse differentiation, maturation and neurite outgrowth [27, 56, 65]. α and β NRXN isoforms have distinct roles in synaptogenesis and synaptic neurotransmission due to separate ligand interactions [13, 27, 56, 63]. The expression of *NRXN1* at glutamatergic and GABAergic synapses supports its role in excitation/inhibition (E/I) dynamics in the brain, which is thought to be a neurobiological mechanism contributing to autism and schizophrenia.

Evidence for altered neural development and E/I dynamics associated with complete loss of *NRXN1* is supported through preclinical cell and animal model studies, although results are heterogeneous. For example, derived induced pluripotent stem cells (iPSCs) from autistic individuals with NRXN1 dels have shown increased calcium signalling and neuronal excitability [8, 9]. In contrast, human-derived isogenic embryonic stem cells (ESC) from individuals with schizophrenia have indicated that *NRXN1* dels lead to reduced presynaptic neurotransmitter release and neuron differentiation [50, 51, 77]. The majority of Nrxn1 murine model studies also point towards altered E/I dynamics in the brain showing altered synaptic strength and neurotransmission [7, 20], with the exception of one study in Nrxn1α models that showed no change in synaptic transmission [51]. White matter microstructural integrity differences have been shown in Nrxn1 knockout rats suggesting altered brain structural development [10]. Studies in various Nrxn1 murine models have also reported behaviours linked to associated neurodevelopmental and neuropsychiatric conditions, e.g. reduced language complexity, communication and social functioning, and increased repetitive behaviours, as well as cognitive differences in reward processing, attention, instrumental learning and spatial dependent learning [2, 6, 24, 36]. Collectively, these findings suggest that *NRXN1* dels may result in aberrant synaptic transmission and neural circuitry potentially related to shifts in E/I balance, which may subsequently contribute to the observed behavioural phenotypes. Neuroimaging may help to characterise the systems-level impact of *NRXN1* deletion, by identifying structural and functional brain alterations linked to neurocognitive and clinical outcomes.

To date there have been no studies providing an in-depth understanding of both neurocognitive and brain-based phenotypes in *NRXN1* del carriers. Here we used a gene-first approach focussed on genetic diagnosis regardless of clinical diagnosis, to assess if a genetic change is related to a clinically recognisable outcome or ‘intermediate phenotype’ such as a cognitive, behavioural or brain-based difference [17]. Examining neuroimaging markers of *NRXN1* del has the potential to identify transdiagnostic neurocognitive or brain phenotypes closely linked to the gene’s biological function. Importantly, the identification of transdiagnostic neuroimaging markers independent of clinical diagnosis offer insights into shared neurobiological mechanisms, specifically synaptic signalling and E/I dynamics. This approach may be complemented by preclinical murine or cell model studies that not only provide mechanistic understanding but are also potential models for drug testing [46]. Alignment of neurocognitive and neuroimaging methodologies has potential to accelerate the development of biologically informed treatments for individuals affected by *NRXN1* dels.

In this exploratory study, we aimed to characterise neurocognition, brain structure and brain function in carriers of *NRXN1* del. We hypothesised that *NRXN1* del carriers would have differences in neurocognitive domains associated with commonly reported clinical outcomes (e.g., autism, ADHD and psychosis). We also hypothesised that *NRXN1* del carriers would show brain functional and structural differences in grey and white matter compared with age and sex matched comparisons. Identifying neurocognitive and brain phenotypes may help to characterise translational markers for conditions such as autism, intellectual disability and psychosis.

## Methods

### Participants

**Fig. 1:**
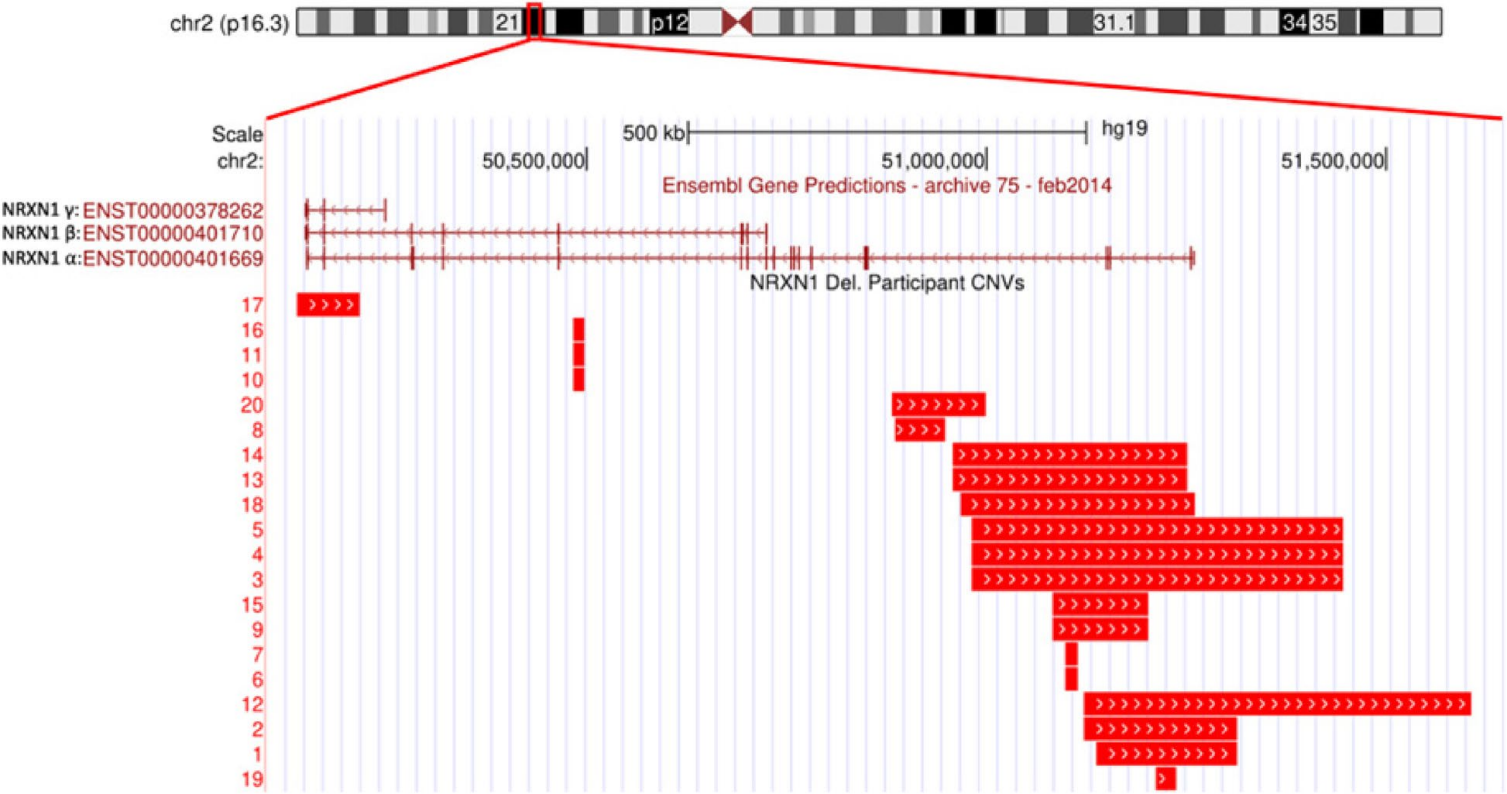
Locations of participant *NRXN1* deletions. Genomic coordinates were mapped to the GRCh37 reference build of the human genome (hg19). The deletions are organised by start position. The three main *NRXN1* transcripts—alpha [ENST00000401669], beta [ENST00000401710], and gamma [ENST00000378262]—are shown above the deletion positions (Ensembl Gene Predictions, archive 75, February 2014). The vertical lines on these transcripts indicate exon positions. This figure was generated using UCSC Genome Browser (http://genome.ucsc.edu)

Forty-two participants, 21 individuals with *NRXN1* dels which included probands referred for genetic testing and first-degree relatives also identified as *NRXN1* del carriers, and 21 age and sex matched neurotypical comparisons were recruited. *NRXN1* del carriers were recruited through the National Centre for Medical Genetics (NCMG) at Children’s Health Ireland, Crumlin (*n* = 17) and an autism genetic database within the Autism and Rare Neurodevelopmental Disorders Research Group at Trinity College Dublin (TCD) (*n* = 4). *NRXN1* del carrier status was identified by whole genome array CGH testing and validation in the NCMG or in the Viapath laboratory, London, UK. Genomic coordinates are given using the Genome Reference Consortium February 2009 build of the human genome (GRCh37/hg 19) (see Table 1 and Fig. 1). Comparison participants were recruited through noticeboards in colleges, activity clubs, internet forums and social media sites. Ethical approval for the study was obtained from St. James’s Hospital/Tallaght University Hospital Research Ethics Committee (REC reference: 2015/03/01). Participants over 18 years provided written consent and parental written consent was provided for those under 18 years. Exclusion criteria for *NRXN1* del carriers were standard MR contraindications for neuroimaging study, and additional pathogenic CNV elsewhere in the genome. Additional exclusion criteria for comparison participants were a history of current neurological or psychiatric disorders, e.g. or current use of psychoactive medication, first-degree relatives with a clinical diagnosis of neurodevelopmental disorder, history of loss of consciousness or serious head injuries. Four *NRXN1* del carriers and their matched comparisons did not complete the MRI protocol due to inability to travel to the neuroimaging centre. A detailed description of the *NRXN1* del cohort and all participant demographics are outlined in Tables 1 and 2 (see Supplementary Material Fig. 1 for breakdown of participants included in each analysis of neurocognitive and neuroimaging data).

**Table 1:** *NRXN1* del carrier genetic and clinical phenotypes

	Sex	Age^1^	Deletion	Size (bp)	Inheritance	Phenotype^2^
Family 1	M	9.8^**^	E1-3 (α)	15,538	Paternal	SLD
	M	52.2	E1-3 (α)	15,538	Unknown	No known diagnosis
Family 2	M	20.3	E1-5 (α)	177,359	De novo	Autism, mild ID
Family 3	M	18.4	E1-5 (α)	192,339	De novo	ASD
Family 4	F	18.6^*^	E1-5 (α)	464,760	Paternal	Schizophrenia, Autism, mild ID
	M	13.7^*^	E1-5 (α)	464,760	Paternal	SLD, Conduct disorder
	M	49.9	E1-5 (α)	464,760	Unknown	Epilepsy
Family 5	M	31.8	E1-5 (α)	484,166	Unknown	No known diagnosis
Family 6	F	33.9^**^	E1-5 (α)	292,159	Maternal	No known diagnosis
Family 7	M	39.8	E3-5 (α)	118,059	Unknown	No known diagnosis
	F	34	E3-5 (α)	118,059	Unknown	No known diagnosis
Family 8	F	13.4^**^	E3-5 (α)	294,102	Paternal	Autism
	M	47.7^**^	E3-5 (α)	294,102	Unknown	Autism
Family 9	M	10.1	E22-25 (α/β)	76,745	Unknown	Autism, ADHD, mild ID
Family 10	M	35.5	Intron-2	24,780	Unknown	No known diagnosis
Family 11	F	43.6	Intron-5	61,342	Maternal	Depression, Anxiety
Family 12	F	33.7	Intron-5	33,893	Unknown	No known diagnosis
Family 13	M	43.9	Intron-5	117,830	Unknown	No known diagnosis
Family 14	M	12.9	Intron-18	12,239	Maternal	ADHD
	F	42.1	Intron-18	12,239	Unknown	No known diagnosis
Family 15	F	47.7	Intron-18	12,239	Unknown	No known diagnosis

*Abbreviations*: *ADHD* Attention deficit hyperactivity disorder, *ID* Intellectual disability, *SLD* Speech and language delay

^1^Age was calculated relative to the date of MRI scan. There was no more than 2 months between neurocognitive testing and performance of the neuroimaging protocol

^2^Phenotypic data was collected from clinical information previously ascertained by families within a clinical setting

^*^Excluded from Resting State MRI analysis due to motion

^**^Did not complete the MRI protocol

**Table 2:** Demographics *NRXN1* del carrier and comparisons

	*NRXN1* del (*n* = 21)	Comparisons (*n* = 21)	
Sex	Male = 13; Female = 8	Male = 13; Female = 8	
Age	Mean: 31.39 ± 14.6 Range: 9.8–52.2	Mean: 31.46 ± 14.6 Range: 10.2–53.6	*p* = 0.988
FSIQ	87.24 ± 10.73 Range: 69–111	107.86 ± 10.48 Range: 90–127	*p* < 0.001

### Neurocognition data acquisition

We assessed neurocognitive functioning with The Wechsler Abbreviated Scale of Intelligence-second edition (WASI-II) (Wechsler, 2011) and a comprehensive cognition battery (CANTAB; http://www.cambridgecognition.com/). The neurocognitive battery consisted of 6 tests used to evaluate 3 cognitive sub-domains; attention/processing speed (Matched to Sample Visual Search (MTS), Rapid Visual Information Processing (RVP)), executive function (Spatial Working Memory (SWM), Stockings of Cambridge (SOC), Intra-Extra Dimensional Shift (IED)) and social cognition (Emotion Recognition Task) (See Methods in Supplementary Materials for more detailed description of tasks). These tasks were selected based on their known associations with *NRXN1 del*-related neuropsychiatric outcomes. The CANTAB ‘clinical’ test mode was selected for each test. Reaction time (RT) was measured to ensure that any observed group differences were not due to motor impairments.

### Neuroimaging data acquisition

All neuroimaging data were acquired on a Philips Achieva 3 T system at Trinity College Dublin.

#### Anatomical data

High-resolution anatomical T1-weighted volumes were acquired using a multi-shot fast field echo sequence (TR = 8.4ms; TE = 8.9ms; 180 slices; voxel size 0.9 × 0.9x0.9mm).

#### Resting state fMRI (RS-fMRI)

Functional MRI data were acquired using a single-shot echo-planar imaging sequence (200 volumes; TR = 2000ms; TE = 25ms; 37 slices; voxel size 3.2 × 3x3mm). Participants were verbally instructed to “stay still and focus on the white cross on the screen”.

#### Diffusion Tensor Imaging (DTI)

DTI data were acquired using a single-shot echo-planar imaging sequence (TE = 73ms, TR = 12,312 ms, FOV = 256, matrix = 128 × 128, 65 slices, 2 × 2x2mm with no gap) Diffusion gradients were applied in 61 directions, with b = 1500 s/mm^2^, and four b = 0s/mm^2^ were also acquired.

### Statistical analysis of neurocognitive data

IBM SPSS Statistics, Version 25 (Armonk, NY: IBM Corp) was used for statistical analysis. Stepwise conditional logistic regression analyses [39] using Cox regression were carried out to account for matched participants between *NRXN1* del and neurotypical comparison groups. Three regression analyses were conducted for each cognitive sub-domain (attention, executive function and social cognition) to determine if performance was associated with *NRXN1* del carrier status. For the attention regression analysis initial variables included: number of correct responses and mean reaction time change on the Match to Sample Visual Search task, as well as mean latency and number of correct responses on the Rapid Visual Information Processing task. For the executive function regression analysis, variables included: strategy and total number of errors for the Spatial Working Memory task; minimum number of moves for the Stockings of Cambridge task; and total errors and completed stage trials for the Intra-Extra Dimensional Shift task. For the social cognition regression analysis, variables included: mean latency and percentage correct responses variables for the Emotion Recognition task. The full list of variables are outlined in Table 3. In addition, to examine whether family members showed similar performance profiles, which could indicate shared genetic or environmental influences, an intraclass correlation analysis was performed to measure familial clustering of neurocognitive performance using a two-way mixed effects model with absolute agreement (see Table 1 for detailed description of *NRXN1* del cohort).

**Table 3:** Performance variables included in cox regression analyses

Cognitive Domain	Task	Performance variables included in regression
Attention	1. Matched to Sample Visual Search 2. Rapid Visual Information Processing	Task 1. Number of correct responses Task 1. Mean reaction time change (ms) Task 2. Mean latency (ms) Task 2. Number of correct responses
Executive Function	1. Spatial Working Memory 2. Stockings of Cambridge 3. Intra-Extra Dimensional Shift	Task 1. Strategy Task 1. Total number of errors Task 2. Minimum number of moves Task 3. Total errors Task 3. Completed stage trials
Social Cognition	1. Emotion Recognition Task	Task 1. Mean latency (ms) Task 1. Percentage correct responses

A paired t-test was performed to examine group differences in reaction time and movement time on the Reaction Time task, accounting for the one-to-one matching of participants between *NRXN1* del and neurotypical comparison groups. This analysis was carried out to identify group differences in baseline motor movements that could account for differences across cognitive domains of interest. Additionally, it could also indicate differences in processing speed. Some of the *NRXN1* del and neurotypical comparison participants did not complete all cognitive subtests for various reasons (e.g. time constraints, disengagement) (see Supplementary Material Fig. 1 for breakdown of participants included in each analysis of neurocognitive data). In such cases, age and sex matched counterparts were removed from the overall analyses.

### RS-fMRI data preprocessing

Data processing was performed using AFNI (http://afni.nimh.nih.gov/afni/), FSL (http://fsl.fmrib.ox.ac.uk), and ANTs (http://stnava.github.io/ANTs) and followed best-practice procedures for minimising the impact of participant motion [59]. Preprocessing comprised (1) volume-based motion-correction, (2) grand-mean scaling, (3) linear and quadratic detrending (4) nuisance signal regression on 24 motion parameters (3 translational and 3 rotational parameters describing participant motion at each TR, their first derivatives, and the squares of each these terms) and 12 nuisance signal regressors, comprising signal from CSF, white matter, and the global signal, their first derivatives, their squares, and the squares of their first derivatives, (5) spatial smoothing (6mm FWHM), and (6) band-pass temporal filtering (0.009–0.1Hz). We opted to perform global signal regression on the basis of evidence that it is the most effective method for reducing the impact of motion and other confounds on functional connectivity metrics [52–54, 59].

Functional-to-anatomical co-registration was performed using bbr (boundary-based registration) [29] as implemented in FSL. Diffeometric normalization of individual anatomical to MNI152 template space (2mm resolution) was performed using ANTs, and the same nonlinear transformation to standard space was applied to the pre-processed functional data.

### Functional connectivity analyses

Given the small and heterogeneous sample, we performed a limited number of targeted analyses aimed primarily at describing the connectivity phenotype associated with *NRXN1* deletions.

**Fig. 2:**
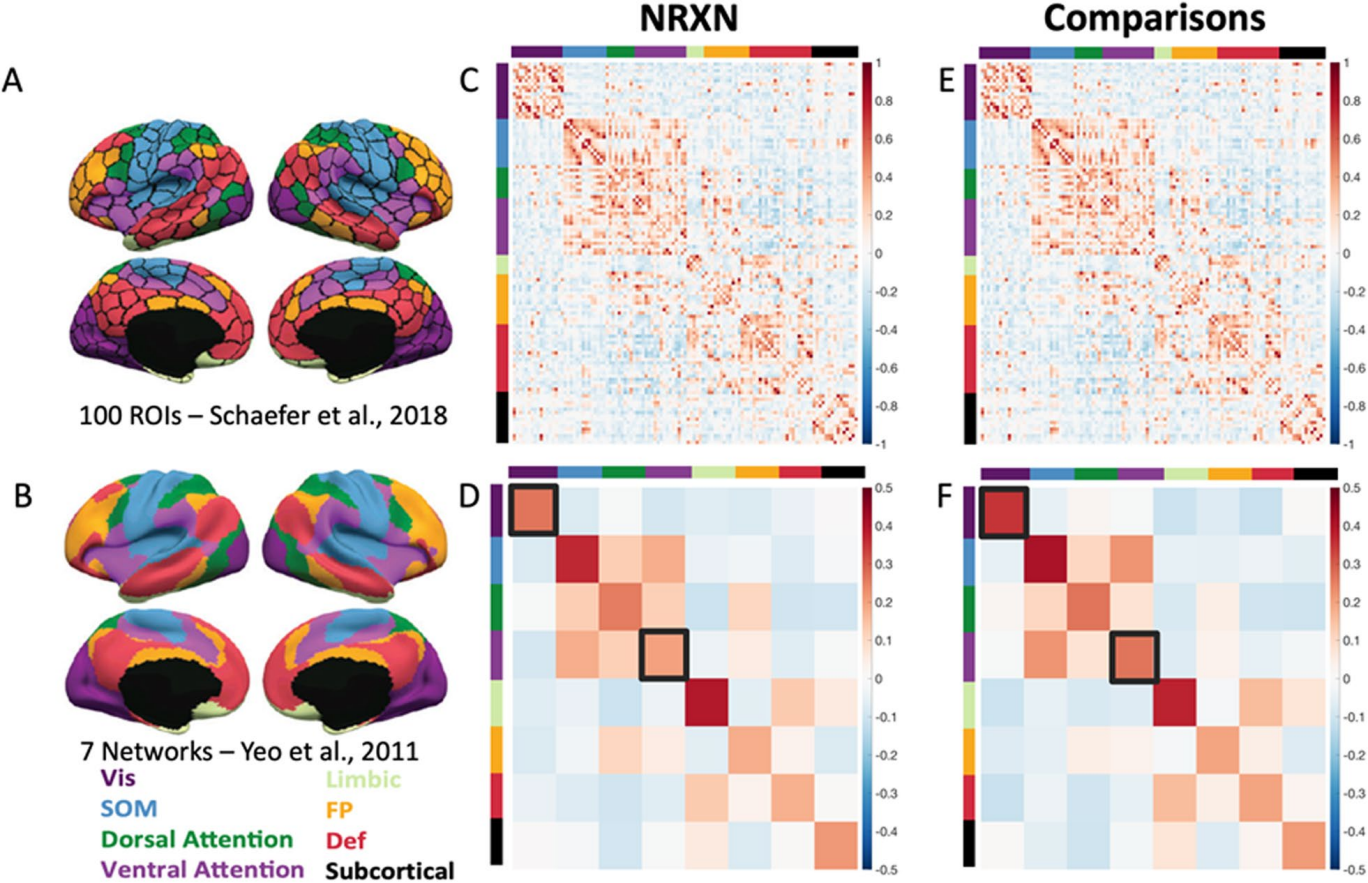
Functional connectivity analyses. **A** 100 Regions of interest (ROIs), derived using a clustering-based parcellation of functional connectivity data [60]. **B** Each of these 100 regions of interest have been assigned to one of seven canonical functional brain networks identified by Yeo et al. [68]: Visual, Somatomotor, Dorsal Attention, Ventral Attention, Limbic, Frontoparietal, and Default Networks, as well as a Subcortical Network with 15 additional ROIs from the Harvard–Oxford subcortical anatomical atlas with FSL (total of 115 ROIs). **C** Mean correlation matrix for *NRXN1* deletion carriers (NRXN) for 115 ROIs and (**D**) 7 networks and subcortical areas. **E** Mean correlation matrix for the neurotypical comparisons (Comparisons) for 115 ROIs and (**F**) 7 networks and subcortical areas. Black squares in (d) and (**F**) indicate significant between-group differences in Visual and Ventral Attention Networks. Abbreviations: Comparisons = Neurotypical Comparison group; Def = Default Network; FP = Frontoparietal Network; Limbic = Limbic Network; NRXN = NRXN1 deletion group; ROI = Region of interest; SOM = Somatomotor Network; Subcortical = Subcortical Network; Vis = Visual Network

To compare functional connectivity between *NRXN1* del carriers and comparisons, we first extracted timeseries for each of 100 regions of interest (ROIs), derived using a clustering-based parcellation of functional connectivity data; (https://github.com/ThomasYeoLab/CBIG/tree/master/stable_projects/brain_parcellation/Schaefer2018_LocalGlobal; Fig. 2A) [60]. Each of these 100 regions of interest have been assigned to one of seven canonical functional brain networks identified by Yeo et al. [68]; Fig. 2B: visual, somatomotor, dorsal attention (DAN), ventral attention (VAN), limbic, frontoparietal, and default networks [68]. The Schaefer parcellation does not include subcortical areas; we added ROIs for hippocampus, amygdala, putamen, caudate, nucleus accumbens, globus pallidus, thalamus, and brainstem, as defined in the Harvard–Oxford subcortical anatomical atlas provided with FSL, resulting in 115 ROIs. The cerebellum was omitted. With the exception of the brainstem, each subcortical ROI had left- and right-hemisphere counterparts. For each ROI the mean BOLD time series was extracted by averaging the signal across voxels within the region.

#### Within-network analysis

To compare mean functional connectivity within each of the seven canonical networks between groups, we first computed the mean Fisher-z-transformed Pearson correlation between all possible pairs of ROIs falling within a network. We then regressed mean functional connectivity on group status (*NRXN1* del; Comparison), controlling for age, sex, and in-scanner motion (mean root mean squared framewise displacement—rmsFD).

#### Voxelwise analysis

To further explore differences in within-network connectivity at the voxel level, we used FSL’s Dual Regression to compute participant-level estimates of functional connectivity within each of the 7 Yeo networks (using the network-level masks available from https://github.com/ThomasYeoLab/CBIG/tree/master/stable_projects/brain_parcellation/Yeo2011_fcMRI_clustering), simultaneously. Next, using FSL’s FEAT, we performed group-level independent samples t-tests to examine the effect of NRXN status (NRXN/Comparison) on functional connectivity within networks identified as differing in mean connectivity between groups (i.e., in the preceding analysis), covarying for sex, age, and motion (mean rms framewise displacement; rmsFD). Regions exhibiting a significant group difference in FC were spatially constrained to occur within the boundaries of the canonical network of interest. Family-wise error was controlled using a non-parametric permutation-based approach, implemented in FSL’s Randomise program, *p* < 0.05, corrected using threshold-free cluster enhancement. A cluster was considered significant if it contained at least 10 connected voxels (80mm3).

#### Edgewise network analysis

Finally, to obtain a broader perspective on potential differences in functional connectivity between groups, we compared functional connectivity between each of the 115 ROIs (i.e., 6555 edges) between groups using the Network Based Statistic [76], covarying for sex, age and motion (mean rmsFD) and using an edge-wise threshold of Z > 3.1 and p (corrected) < 0.05.

### White matter structural architecture analysis

DTI data were pre-processed using ExploreDTI (v4.8.4) software (www.exploredti.com/) and FA, MD, RD, and AD diffusion images were extracted. Tract Based Spatial Statistics (TBSS) was carried out using FSL software (http://www.fmrib.ox.ac.uk/fsl/) (see Methods in Supplementary Materials for more detailed description of DTI processing and TBSS analysis).

Voxel-wise statistics were performed using the randomize permutation-based inference tool for nonparametric statistical thresholding within FSL (Winkler et al., 2014). FA, MD, RD and AD measures were compared between *NRXN1* del and comparison groups, accounting for age and gender. Results were thresholded to a *p*-value of < 0.05, corrected for multiple comparisons using the threshold-free cluster enhancement (TFCE). (See Supplementary Material Table 12 for a full list of ROI labels, centroid coordinates, and network assignments).

### Grey matter structural analysis

FreeSurfer image analysis suite (v6.0.0) software (http://surfer.nmr.mgh.harvard.edu/) was used to perform automated volumetric segmentation and cortical reconstruction of T1-weighted anatomical images (see Methods in Supplementary Materials for more detailed description of processing and analysis). Regional differences in brain volume, surface area and cortical thickness metrics were compared between NRXN1 and comparison groups using t-tests, with age and sex as covariates, and estimated total intracranial volume (eTIV) for volume and surface area comparisons only. Results were thresholded to a *p*-value of < 0.05, corrected for multiple comparisons using family wise error (FWE) correction. For volumetric analysis, global measures of total eTIV, total cortical volume, total subcortical volume and total white matter volume were compared between NRXN1 and comparison groups, using SPSS (Version 25).

### Exploratory brain-behaviour analyses

To explore potential brain-behaviour links, we performed an additional set of group analyses examining the association between functional connectivity and the behavioural variable that showed a significant effect of NRXN status: Spatial Working Memory (total errors). These analyses were conducted separately within each group (NRXN, Comparisons), covarying for sex, age, IQ, and motion (mean rmsFD). To reduce the number of statistical tests, we restricted this analysis to networks (in the ROI-ROI analyses), regions (in the voxelwise analyses), and edges (averaged) that showed significant group differences.

## Results

### Neurocognitive data analysis

#### Reaction time

There was no significant between-group difference in reaction time for the 1-choice (t (17) = 1.584, *p* = 0.132) or 5-choice test (t (17) = 1.49, *p* = 0.154). Similarly, there was no difference in movement time for either the 1-choice (t (17) = −0.414, *p* = 0.684) or 5-choice tests (t (17) = 0.52, *p* = 0.61).

#### Attention

None of the variables in the model were significantly associated with the presence of a *NRXN1* del, *p* > 0.05.

#### Executive function

Total number of errors (SWM) was significantly associated with *NRXN1* carrier status, Exp (B) = 1.104, CI 95% = 1.008–1.208, *p* = 0.032. This finding indicates that individuals with deficits in SWM are more likely to have a *NRXN1* del. None of the other variables were significant, *p* > 0.05.

#### Social cognition

None of the variables in the model were significantly associated with the presence of a *NRXN1* del, *p* > 0.05.

#### Intraclass coefficient analysis

There was no significant intraclass correlation in behavioural performance for any variable across the participants.

### Neuroimaging data analysis

#### Participants

All datasets were visually inspected for artefact, and participants exhibiting motion > 0.15mm rmsFD were excluded from analyses (see Supplementary Materials Table 4 for participant motion reported as rmsFD) [32]. Two participants were excluded from the NRXN1 del group, and two were excluded from the comparison group. As a result of these exclusions, no siblings remained in the imaging sample. The final sample used for rs-fMRI imaging analyses included 13 *NRXN1* del carriers (mean age = 37.2; range 10.1–52.2; 5 female) and 15 Comparisons (mean age = 34.2; range 10.2–53.6; 6 female). There was no difference between groups in terms of mean rmsFD (mean NRXN = 0.1008, mean Comparisons = 0.0909; t(25) = 0.799, *p* = 0.431).

#### Functional connectivity

The upper panel of Fig. 1 shows the mean correlation matrix for *NRXN1* deletion carriers (C) and comparisons (E) for the 115 ROIs. The lower panel of Fig. 1 shows the mean within- and between-network correlations for each of the seven canonical functional brain networks identified by Yeo et al. [68]: visual, somatomotor, dorsal attention, ventral attention, limbic, frontoparietal, and default networks, as well as subcortical regions, for *NRXN1* deletion carriers (D) and comparisons (F).

##### Within-network analysis results

**Fig. 3:**
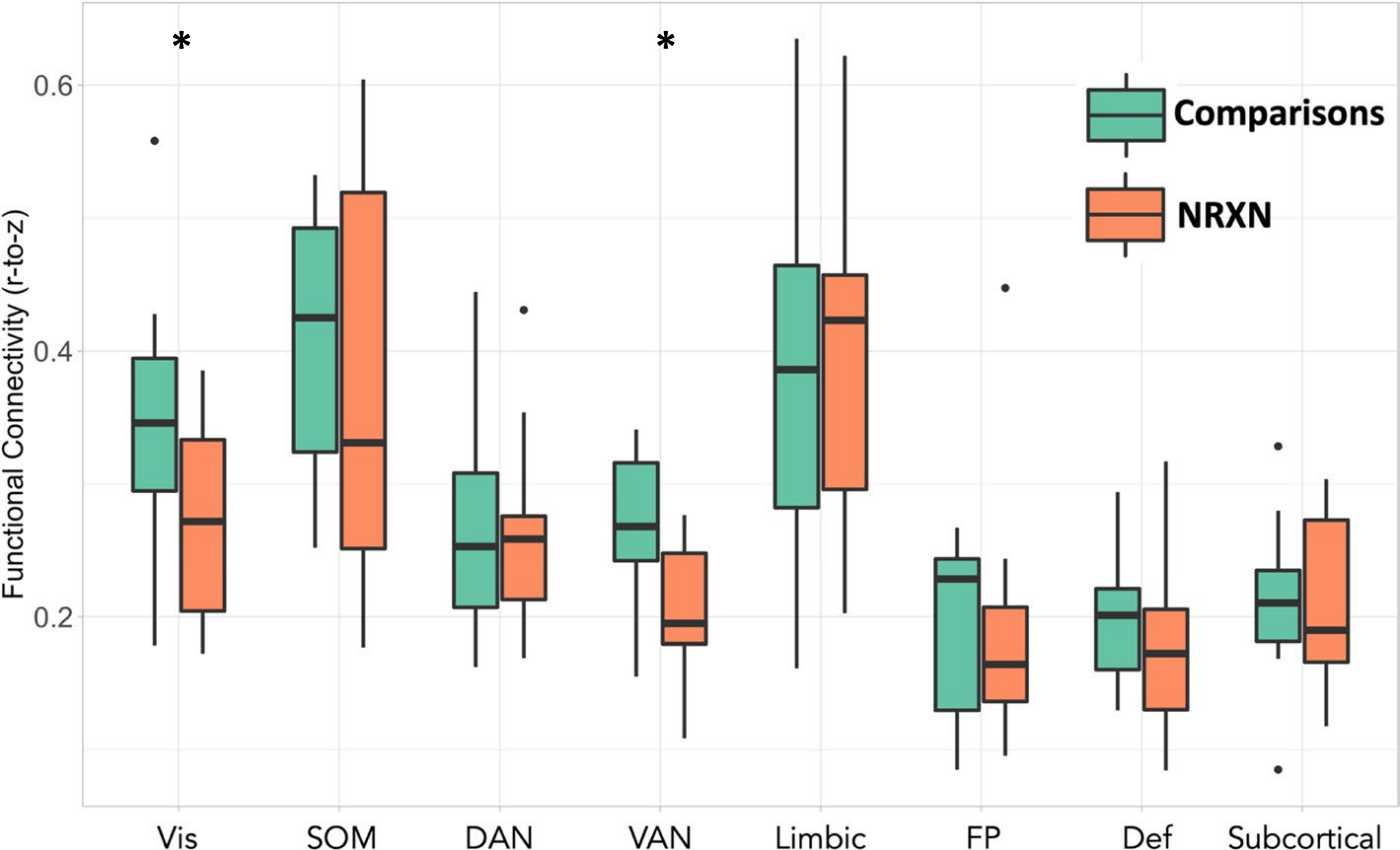
Mean within-network functional connectivity within the seven cortical functional brain networks and the subcortical regions. The group difference in within-network functional connectivity (mean Fisher-z-transformed Pearson correlation between all possible pairs of ROIs falling within a network) was significant for the Visual Network and the Ventral Attention Network. *indicates significant between-group differences (*p* < 0.05). Abbreviations: Comparisons = Neurotypical Comparison group; DAN = Dorsal Attention Network; Def = Default Network; FP = Frontoparietal Network; Limbic = Limbic Network; NRXN = NRXN1 deletion group; SOM = Somatomotor Network; Subcortical = Subcortical Network; VAN = Ventral Attention Network; Vis = Visual Network

To control the number of statistical comparisons performed, we compared only mean *within*-network connectivity between groups (i.e., shown on the diagonal of Fig. 2D and F). Within-network connectivity was significantly different between *NRXN1* del and neurotypical comparison groups for the Visual network (β = −0.075, SE = 0.032, 95% CI [−0.142, −0.009], *p* = 0.028) and Ventral Attention network (β = −0.061, SE = 0.022, 95 CI = [−0.106, −0.015], *p* = 0.011), controlling for in-scanner movement (mFD), age, and sex (see Tables 4–11 in Supplementary Materials for detailed within-network connectivity statistical analysis results). Within-network connectivity was weaker in the *NRXN1* del group, relative to neurotypical comparisons (Fig. 3).

##### Voxelwise analysis results

**Fig. 4:**
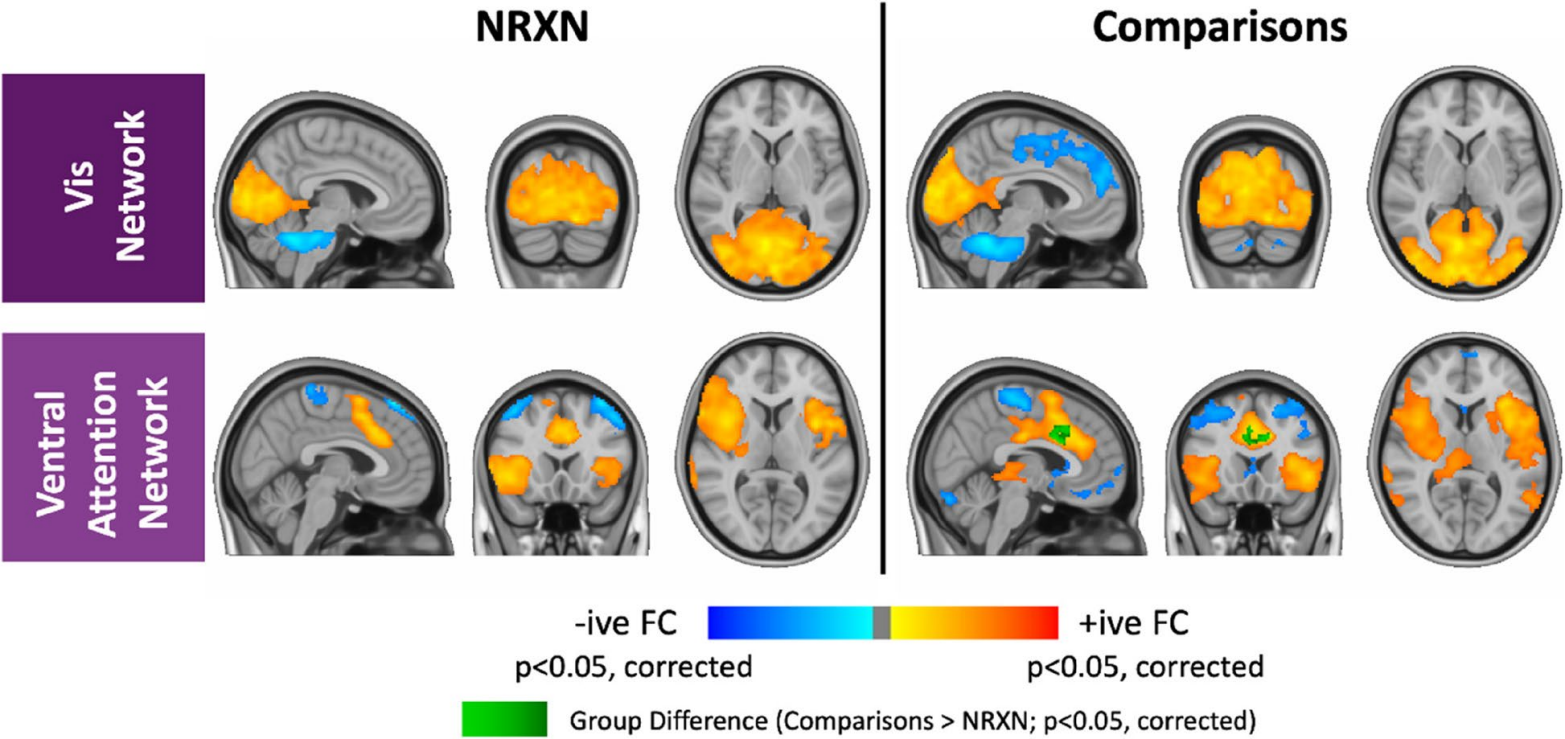
Voxelwise functional connectivity maps. Positive (orange-yellow) and negative (blue-light blue) functional connectivity for the *NRXN1* del (NRXN) and Neurotypical Comparison (Comparisons) groups, within the Visual and Ventral Attention Networks, obtained using Dual Regresssion and displayed as voxelwise t-values (from one-sample voxelwise t-tests). Familywise error was controlled at *p* < 0.05 using a non-parametric permutation-based approach (FSL’s Randomise, with threshold-free cluster enhancement). A cluster was considered significant if it contained at least 10 connected voxels (80mm^3^). Only the Ventral Attention Network exhibited a significant group different in voxelwise functional connectivity (in an independent groups t-test), with *NRXN1* deletion carriers showing weaker functional connectivity in the dorsal anterior cingulate cortex (dACC), relative to neurotypical comparisons. Abbreviations: Comparisons = Neurotypical Comparison group; NRXN = NRXN1 deletion group; -ive FC = negative functional connectivity; + ive FC = positive functional connectivity

Figure 4 shows the voxelwise functional connectivity maps for the *NRXN1* del carrier and neurotypical comparison groups for the Visual (upper panel) and Ventral Attention (lower panel) networks. Positive functional connectivity is shown in orange; negative functional connectivity is shown in blue. No significant voxelwise differences were detected for the Visual network. For the Ventral Attention network, *NRXN1* del carriers exhibited significantly weaker functional connectivity in bilateral dorsal anterior cingulate gyrus (dACC), relative to comparisons (*p* < 0.05, TFCE corrected) (shown in green in the lower right panel of Fig. 4). Given the small sample size, it is important to acknowledge the possibility of both Type I and Type II errors in these voxelwise analyses.

##### Edgewise network analysis results

**Fig. 5:**
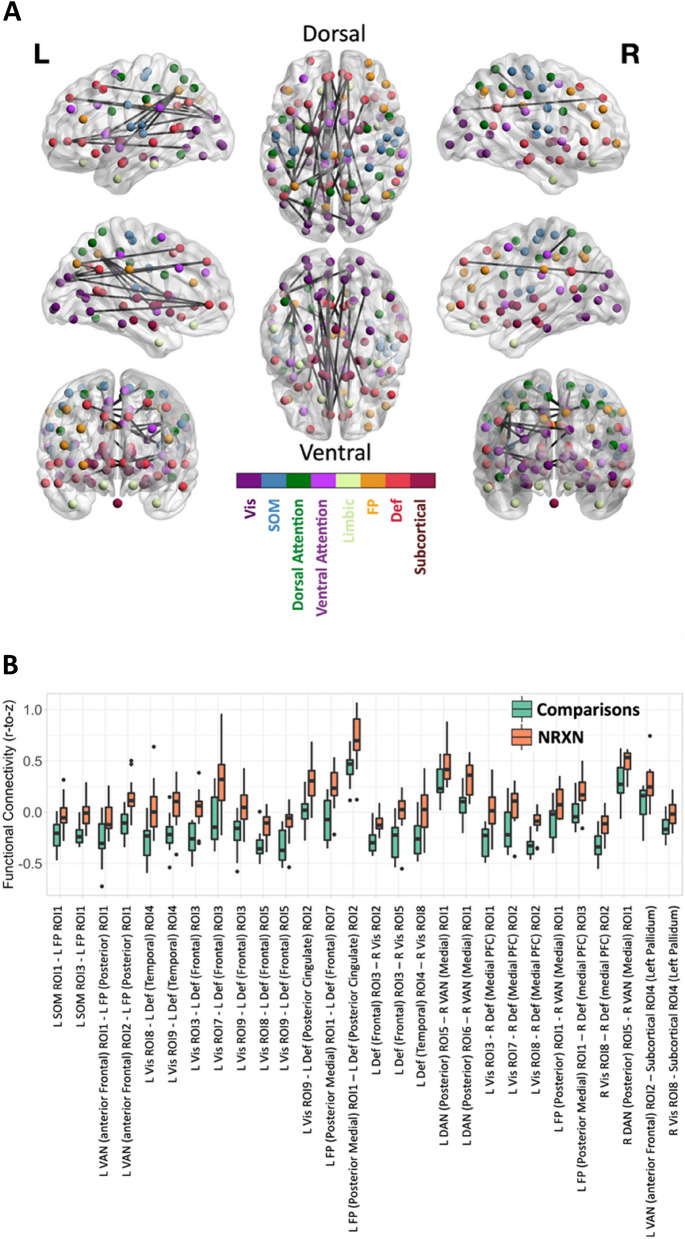
Edgewise network analysis using Network Based Statistic. **A** Edges (ROI-ROI connections, shown in black) showing a significant effect of NRXN status (*NRXN1* del > Comparisons) in the Network Based Statistic analysis. ROIs are colour-coded according to their network membership. 28 connections showed a significant effect of group status, all *NRXN1*del carriers > Comparisons; 14/28 were between ROIs in the left hemisphere, 10 between contralateral ROIs, 2 cortico-subcortical, 2 between ROIs located in the right hemisphere. **B** The boxplots show the median and interquartile range for functional connectivity (i.e., mean Fisher-z-transformed Pearson correlation) within each group (Orange = NRXN1 del carriers; Green = Neurotypical Comparisons) for edges (i.e., ROI-ROI connections) showing significant effect of group status (i.e., *NRXN1* del > Comparisons) in the Network Based Statistic analysis using an edge-wise threshold of Z > 3.1 and corrected p (corrected) < 0.05. Abbreviations: L = Left; R = Right; ROI = Region of Interest; Vis = Visual Network; SOM = Somatomotor Network; VAN = Ventral Attention Network; DAN = Dorsal Attention Network; FP = Frontoparietal Network; Def = Default Network; PFC = Prefrontal Cortex. A full list of ROI labels, centroid coordinates, and network assignments is provided in Table 12 in Supplementary Materials

Again, acknowledging the limitations posed by the small sample, we also compared functional connectivity between each pairing of the 115 ROIs between groups using the Network Based Statistic [76], to obtain a broader perspective on the impact of *NRXN1* deletion carrier status on functional connectivity. Figure 5 illustrates the result of this analysis, which compared functional connectivity between groups while controlling for rms FD, age, and sex. Twenty-eight connections showed a significant effect of group status, specifically *NRXN1* del carriers > neurotypical comparisons (*p* < 0.05, corrected). Inspection of the specific connections affected reveals that 14/28 of these connections were between ROIs located in the left hemisphere, a further 10 were between contralateral ROIs (i.e., left-to-right), two were cortico-subcortical, and two were between ROIs located in the right hemisphere. Figure 5 shows that 23/28 were functional connections that were negative amongst neurotypical comparisons, but less strongly negative, or weakly positive, amongst *NRXN1* del carriers, suggests poorer segregation of networks in *NRXN1* del carriers. A substantial proportion of these were negative correlations between ROIs located in the Visual network and ROIs located in the Default Network. Two of the connections exhibiting a group difference in positive functional connectivity (*NRXN1* del > Comparisons) were between ROIs located in the DAN and VAN, indicating stronger relationships between these networks in NRXN1 deletion carriers.

### White matter structural architecture

There were no statistically significant differences in diffusion measures of FA, MD, RD or AD between *NRXN1* del and neurotypical comparison groups, when age and sex were included as covariates (*p* > 0.05, TFCE corrected).

### Grey matter structure

There were no statistically significant differences between *NRXN1* del and neurotypical comparison groups in regional comparisons of cortical thickness, brain volume or surface area, when age and sex were included as covariates, and eTIV for volume and surface area (*p* > 0.05, FWE corrected). There were no statistically significant differences between *NRXN1* del and neurotypical comparison groups in global volumetric comparisons of eTIV, total cortical volume, total subcortical volume, and total white matter volume (all *p* > 0.05).

### Exploratory brain-behaviour analyses

We explored associations between Spatial Working Memory performance (total errors) and ROI-ROI analysis networks, voxelwise analysis regions and edgewise network analysis edges showing significant group differences. No significant relationships were detected (all *p* > 0.05).

## Discussion

This is the first study, to our knowledge, to characterise neurocognition and brain structure and function in a cohort of *NRXN1* del carriers. Performance on a SWM task, with significant executive function demands, predicted *NRXN1* del carrier status. No differences were observed between the groups on the remaining cognitive tasks tested, although there was a tentative reduction in performance on a task of social cognition. Neuroimaging identified significant differences in resting state functional connectivity in the visual and ventral attention networks between *NRXN1* del carriers and comparisons, and suggested poorer segregation of neural networks, principally between the visual network and both the default mode and fronto-parietal networks. Grey and white matter structural analysis revealed no differences between groups.

### Neurocognitive findings grounded in ND-CNV and neuropsychiatric condition literature

There have been limited studies of cognitive profiles carriers of *NRXN1* del. Two studies have reported on clinically ascertained cohorts, and two studies have reported on ND-CNV carriers from the UKBiobank, a population-based cohort. In relation to clinically ascertained cohorts, one study of five children reported mildly reduced cognitive ability in carriers (below-average or lower limit of average cognitive ability) [3], whereas wide variability in cognitive ability was found in the current study of children and adults ranged from below average to above average (FSIQ = 69–111). A second study of carriers of different ND-CNVs identified reduced SWM in a small cohort of 14 *NRXN1* del carriers (z = −1.07), however this was not significantly different to non-carrier sibling comparisons [15]. Neither this study nor Chawner et al. [15] identified *NRXN1* del associated differences in spatial planning (Stockings of Cambridge task), visual attention, or sustained attention (rapid visual processing task), and we also found no differences in social cognition (emotion recognition task). It is difficult to compare results with the previous study due to study design differences. For example, the comparison groups and participant age ranges were different, and the *NRXN1* del cohort in the current study included adults as well as parent carriers without a confirmed clinical diagnosis of a neurodevelopmental condition (see Table 1). Further, although these tasks were selected to measure autism and ADHD-related neurocognitive domains, given the association with *NRXN1* del [48], the lack of findings may also suggest a limitation in power to detect any performance differences in the neurocognitive tasks.

Carriers of multiple different ND-CNV were examined from the UKBiobank, a population-based cohort, and indicated differences in simple and complex processing speed in carriers of *NRXN1* del [33]. While different neurocognitive tasks were implemented in the current study, the findings did not suggest that *NRXN1* del was associated with reduced processing speed, as measured by a reaction time task or reaction time variables across different tasks. The discrepancies in age and ascertainment between the UKBiobank and the current study must be acknowledged when comparing findings between a population-based cohort and a clinically ascertained cohort.

Interestingly, poorer SWM performance is reported in other ND-CNV, such as 22q11.2 deletions [49] and deletions and duplications of 1q21.2 [42], although age effects have been reported. In this context, SWM may represent a heritable phenotype under genetic control. Further support for this is evidenced by reports of poorer SWM in individuals with neurodevelopmental and neuropsychiatric conditions and their first-degree relatives, including autism [61, 73], ADHD [67, 71], and first episode psychosis [19, 40], although this has not been universally replicated in first episode psychosis [44].

### Altered brain function in carriers of *NRXN1* deletions

We also report the first fMRI findings in *NRXN1* del carriers, showing weaker within network connectivity in visual and ventral attention networks in *NRXN1* del carriers relative to comparisons, and weaker voxelwise functional connectivity in bilateral dorsal anterior cingulate gyrus (dACC).

Our result of weaker voxelwise functional connectivity in the bilateral dACC gyrus needs to be cautiously interpreted given the sample size. Notwithstanding, the dACC is implicated in a range of cognitive and motor functions relevant to autism and ADHD that could be relevant to clinical outcomes in *NRXN1* del carriers such as conflict monitoring, social evaluation, reward-based learning, inhibitory control, consciousness and pain monitoring and ‘cold’ executive functions [1, 35, 58]. The dACC has been associated with co-occurring anxiety and autism [11]. Separately, individuals with first episode schizophrenia, bipolar and psychotic bipolar, showed reduced functional connectivity between the dACC and medial frontal gyrus, medial temporal gyrus, caudate and cerebellum suggesting cross-disorder connectivity of the dACC [41]. Disorder-specific ACC functional correlations with psychotic symptoms were also reported, left dACC and the right caudate in psychotic bipolar, and caudal ACC and DMN and a visual processing region for first episode schizophrenia. We did not find any correlation between the dACC connectivity and cognitive performance, nor did we measure autism, ADHD or schizophrenia traits in our study. Further investigation of this brain region in relation to cognitive function and clinical features is necessary.

Findings from the Network Based Statistics analysis in the present study suggested poorer segregation of the visual and default networks and the visual and frontoparietal networks in the *NRXN1* del carriers. Fronto-temporal and occipito-temporal dysconnectivity or weaker associations between these subnetworks has been reported in schizophrenia [76]. This contrasts with recent findings in autism using the Autism Brain Imaging Data Exchange (ABIDE) dataset which suggested greater segregation and less connectivity in autism [5]. Increased functional connectivity across nodes within the cerebellum, thalamus, and cerebral cortex have also been proposed as a precursor to the onset of psychosis and a feature present in schizophrenia [14]. This suggests that these hyperconnectivity patterns might serve as a neural marker for psychosis. Although we did not include cerebellum within our resting state analysis, gene expression data suggest that NRXN1 is highly expressed in the cerebellum, pointing towards further investigation of functional connectivity of this brain region.

We observed no differences in grey or white matter structure between *NRXN1* del carriers compared with age and sex matched comparisons This has not been assessed previously in *NRXN1* del carriers. A common variant in the 3’UTR of the *NRXN1* gene at a putative microRNA site was associated with whole brain and frontal lobe white matter volumes in healthy individuals [72]. Separately, an intronic SNP (rs12467877) was reported to be associated with enlargement of the Temporal Horns of the Lateral Ventricles in psychosis, psychosis outcomes, reduced hippocampal volume and enlargement of the choroid plexus and caudate [4]. Many of these studies were not replicated and were likely underpowered to evaluate the effect of common genetic variation at the *NRXN1* locus.

Although structural differences have been reported in neurodevelopmental conditions, findings are also variable. Age and sexeffects likely contribute to this variability in structural differences [12]. For example, studies of autism indicate highly heterogeneous structural differences [62, 75], and mega-analyses in ADHD report structural brain differences in children, but not adolescents or adults [25, 31]. In the current study, no statistically significant structural differences were observed between *NRXN1* del carriers and neurotypical comparisons. This may reflect several factors, including limited statistical power, age-related variability, clinical heterogeneity within the *NRXN1* del group, or subtle structural effects (e.g. on white matter or cortical organization) that were not detectable. In contrast, functional connectivity differences were observed, suggesting that functional alterations may be more readily detectable.

### What is the relationship with animal models and stem cell work?

Gene first approaches potentially allow for comparison of phenotypes with preclinical models provide insights into neurobiological mechanisms [46]. However, to date, studies of Nrxn1 animal model phenotypes have not been well aligned with human studies [24, 28, 74]. Behaviourally, *Nrxn1* homozygous murine knockouts show altered social approach and reduced social investigation [28, 36], however, spatial working memory has not been reported as a phenotype [28]. We also did not examine repetitive behaviours or reward processing which have been implicated in animal model studies [2, 24].

Electrophysiological studies indicate brain functional differences, with Nrxn1α knockout mice show reduced excitatory synaptic transmission in the hippocampal CA1 region [24], and from the dorsal medial prefrontal cortex to the amygdala [7]. In contrast to our lack of structural white matter differences, one study in Nrxn1 knockout rats revealed altered microstructural integrity in the left capsule and right neocortex, but no difference in neurite density [10]. While studies suggest brain and behavioural outcomes in Nrxn1 animal models, aligning further preclinical and clinical research is necessary to identify potential cross-species biomarkers [46].

We separately reported evidence of increased calcium transients and excitability in *NRXN1*α +/- cortical neurons derived from autistic *NRXN1* del carriers associated with upregulation of glutamatergic synapses (GRIN1, GRIN3B, SHANK1) and ion channels/transporter activity (SLC17A6, CACNG3, CACNA1A) in the transcriptomes of the cortical neurons [8, 9]. Others have shown a different phenotype of synaptic impairments in iPSC derived cortical neurons from individuals with SCZ [50, 51]. Synaptic deficits were not identified in murine Nrxn1 knockout models nor in mouse neurons carrying the heterozygous *Nrxn1* del Allele murine suggesting that the synaptic phenotypes are unique to the human neuronal context [50]. It has been suggested that human are more likely to exert their effects through transcriptomic changes, possibly including the generation of aberrant isoforms [69]. While it seems plausible that altered neuronal excitability may be associated with a dysconnectivity phenotype in carriers of *NRXN1* del, the participants in the current study and iPSC studies do not all overlap, therefore it is not possible to draw direct conclusions from these studies regarding underlying mechanisms.

### Limitations

The sample size in the current study was small due to the relative rarity of *NRXN1* del. The present study also reports outcomes in a subset of individuals with higher cognitive ability for whom neurocognitive and neuroimaging procedures were accessible. This study is exploratory and largely descriptive, and aimed to use these preliminary findings to generate future hypotheses-driven research in *NRXN1* del. Given that small sample size and the exploratory nature of this study, we acknowledge the limitations of the stepwise approach used for the regression analysis of neurocognitive data. The absence of an association between the rsFC findings and neurocognitive findings suggests that associations with clinical traits may not have been observed. It is likely that larger sample sizes are required to investigate these associations.

While approximately half of the participants had clinical diagnoses of a neurodevelopmental and/or neuropsychiatric condition, in-depth clinical phenotyping was not included here nor were trait-based measures. Future studies aim to provide more in-depth behavioural assessment of clinical phenotypes [17].

Individuals with additional pathogenic CNV elsewhere in the genome were excluded, however, we did not investigate the effects of polygenic variants or rare SNV elsewhere in the genome. This will be investigated in the context of the AIMS-2-TRIALS Synaptic Gene Study [17]. Finally, whole genome sequencing was not conducted for the comparison group to examine rare pathogenic CNV, or SNV, as it was beyond the scope of the current study, therefore, it is unknown whether the any individuals from this comparison group may carry a neurodevelopmental-related genetic variant.

## Conclusions and future directions

Using a gene-first strategy can potentially link brain and behavioural phenotypes more closely to the underlying neurobiology across clinical diagnostic categories. This study represents a first step to exploring brain-based biomarkers in *NRXN1* del carriers, by identifying altered resting state functional connectivity patterns associated with *NRXN1* deletions in a clinically ascertained cohort. Future research should examine functional and structural brain development in carriers of *NRXN1* del identified through population-based cohorts, such as the UKBiobank, to determine whether findings are reproducible across cohorts with different ascertainment. Larger, harmonised collaborative datasets and transnational collaborations will be required for replication and further investigation.

## Supplementary Information

Supplementary Material 1.

## Data Availability

The data that support the findings of this study are available from the authors but restrictions apply to the availability of these data.
