# Supplementary material for: Evidence of neurocognitive and resting state functional connectivity differences in carriers of NRXN1 deletions
Source: J Neurodev Disord. 2025 Oct 15;17:62. doi: 10.1186/s11689-025-09625-5 (PMC12522947; doi:10.1186/s11689-025-09625-5)
Supplement: Supplementary file 1 — Supplementary Material 1. [file 11689_2025_9625_MOESM1_ESM.docx]

## Supplementary Materials:

## Supplemental Methods

**Participants**

Figure 1. Flow chart of participants included in neurocognition and MRI data collection and each analysis. Two participants were removed from each group following processing of rs-fMRI data. One participant was removed from NRXN1 del group following processing of DTI and T1 data.

**CANTAB tasks**

### Attention

*Matched to Sample Visual Search (MTS)*

The MTS test involves attention and has a processing speed/accuracy trade-off. Participants are required to match an abstract shape in the centre of the screen with 1 of 2, 4, 6 or 8 shapes, which appear around the edge of the screen. Only one shape is correct, and the remainder are composed of juggled or distractor elements. Performance is measured by two variables; number of correct responses and mean reaction time change (the mean additional time it takes to make a correct response with 8 stimuli rather than 2 stimuli).

*Rapid Visual Information Processing (RVP)*

The RVP is a test of visual sustained attention. Numbers (2-9) appear at random inside the box at a rate of 100 numbers per minute. Participants are instructed to respond to target sequences (2-4-6, 3-5-7, 4-6-8). Two variables, mean latency and number of correct responses are extracted to evaluate behavioural performance.

### Executive function

*Spatial Working Memory (SWM)*

Retention, recall, and manipulation of visuospatial information are required for the SWM test. The aim of this test is for the participant to collect hidden tokens inside a number of boxes. Tokens do not appear in the same box twice. The number of boxes presented increases to 4, 6 and 8 in order to increase the difficulty of the test. Behavioural performance was evaluated using two outcome variables, strategy and total number of errors.

*Stocking of Cambridge (SOC)*

The SOC test assesses spatial planning and spatial working memory ability. Participants are required to adapt the position of circles to match the pattern in the upper display. The test may require 2, 3, 4 or 5 moves in order to complete the pattern representing an increase in difficulty. The minimum number of moves required to complete the patterns provides the outcome variable to evaluate spatial planning.

*Intra-Extra Dimensional Shift (IED)*

This test provides a measure of cognitive flexibility. Four white boxes are displayed on screen and stimuli consisting of two dimensions; coloured shapes and white lines are presented in 2 of the boxes. Participants must learn which stimulus is correct by touching it and continue until the criterion is reached. The test consists of 9 blocks and participants proceed through the test by making 6 consecutive correct responses in line with particular criteria. If the participant fails to satisfy a set of criteria after 50 trials, the test is terminated. In block 6, the intradimensional shift occurs. New compound stimuli are presented and participants must continue to attend to the dimension of the coloured shapes to learn which of the new figures is correct. In block 8, the extradimensional shift is introduced where the participants have to attend to the previously irrelevant stimulus (the white lines) in order to satisfy criteria. Total errors and completed stage trials were used as outcome variables.

### Social cognition

*Emotion Recognition Task (ERT)*

The ERT test measures participants’ ability to identify emotions in facial expressions. Two blocks of 90 stimuli are presented which are computer morphed images derived from the facial features of real individuals. A face depicting the particular emotion is presented for 200ms before participants must select which 1 of 6 emotions (happiness, sadness, anger, fear, surprise and disgust) was expressed. Mean latency and the percentage of correct responses are extracted to measure performance.

**White matter Structural architecture Analysis**

*Pre-processing of DTI Data*

16 *NRXN1* deletion carriers and 19 comparisons were included in the final DTI analysis. 1 NRXN1 deletion carrier was excluded from analysis due to a brain anomaly. DTI data were pre-processed using ExploreDTI software (v4.8.6) ([www.exploredti.com/](http://www.exploredti.com/)) (Leemans et al. 2009a). Data quality checks were performed, including visual inspection of correct orientation of gradient components and checking for gross artefacts. The data were corrected for motion correction, eddy current induced geometric distortions and for echo-planar imaging (EPI) deformations by co-registering and resampling to each subjects’ T1-weighted anatomical image (Irfangolu et al., 2012). The B-matrix rotation was performed within this step to reorient the data appropriately (Leemans et al., 2009b). The robust extraction of kurtosis indices with linear estimation (REKINDLE) approach was utilized when the tensor model was applied to the data (Tax et al., 2015). Fractional anisotropy (FA), mean diffusivity (MD), radial diffusivity (RD) and axial diffusivity (AD) were then extracted from the diffusion data preprocessed using ExploreDTI.

*TBSS Analysis*

FSL software (<http://www.fmrib.ox.ac.uk/fsl/>) (Smith et al., 2004) was employed to perform whole brain voxel-wise analysis of white matter using Tract Based Spatial Statistics (TBSS) (Smith et al., 2006). A nonlinear registration was performed to align each subject’s FA image to a target template image (FMRIB58_FA standard space image). The target image was then affine aligned into 1x1x1mm MNI152 space. Each subject’s FA image was warped into MNI space by combining the non-linear transformation to the chosen target template FA image with the affine transformation to MNI space. FA images from all subjects were then averaged to create the mean FA image and thinned to create a mean FA skeleton. A threshold of 0.2 was then applied to the FA skeleton. Each subject's aligned FA data was projected onto this skeleton and voxelwise statistical analysis was performed. The same non-linear transformation and skeleton projection used for the FA images was applied to the MD, RD and AD diffusion measures.

*Statistical Analysis*

Voxel-wise statistical analysis was performed using the randomize permutation-based inference tool for nonparametric statistical thresholding within FSL (Winkler et al., 2014). FA, MD, RD, and AD measures were compared between NRXN1 and comparison groups using t-tests, with age and gender included as covariates. Five thousand permutations were applied. Results were thresholded to a p value of <0.05 corrected for multiple comparisons using the threshold-free cluster enhancement (TFCE) option from the randomize permutation testing tool in FSL.

**Grey Matter Structural Analysis**

*FreeSurfer Analysis*

16 NRXN1 deletion carriers and 19 comparisons were included in the final structural analysis. One NRXN1 deletion carrier was excluded from analysis due to a brain anomaly. FreeSurfer image analysis suite (v6.0.0) software (http://surfer.nmr.mgh.harvard.edu/) was used to perform automated volumetric segmentation and cortical reconstruction of T1-weighted anatomical images. Processing included motion correction, removal of non-brain tissue (Segonne et al., 2004), automated Talairach transformation, segmentation of the subcortical white matter and deep gray matter volumetric structures (including hippocampus, amygdala, caudate, putamen, ventricles) (Fischl et al., 2002; Fischl et al., 2004a) intensity normalization (Sled et al., 1998), tessellation of the gray matter white matter boundary, automated topology correction (Fischl et al., 2001; Segonne et al., 2007), and surface deformation. Cortical thickness (CT) was computed as the distance from the gray-white matter boundary to the gray matter /cerebrospinal fluid boundary at each vertex on the tessellated surface. Vertex-based estimates of SA were derived.

*Statistical Analysis*

Group comparisons of regional volume, surface area and cortical thickness were performed between *NRXN1* del and comparison groups, with age and gender included as covariates, and estimated total intracranial volume (eTIV) for volume and surface area comparisons only. Results were thresholded to a p-value of <0.05, corrected for multiple comparisons using family wise error (FWE) correction.

## Supplemental Results

**Neurocognition Results**

*Cox Regression Analysis Results*

 Table 1. Attention Cox regression model results.

|  | | **B** | **SE** | **Wald** | **df** | **Sig.** | **Exp(B)** | **95.0% CI for Exp(B)** | |
| --- | --- | --- | --- | --- | --- | --- | --- | --- | --- |
|  |  |  |  |  |  |  |  | **Lower** | **Upper** |
| **Step 1*** | **MTS percentage correct** | -.421 | .232 | 3.312 | 1 | .069 | .656 | .417 | 1.033 |

*Variables not in the equation: MTS Mean reaction time change (ms), RVP Mean latency (ms), RVP Number of correct responses

 Table 2. Executive function Cox regression model results.

|  | | **B** | **SE** | **Wald** | **df** | **Sig.** | **Exp(B)** | **95.0% CI for Exp(B)** | |
| --- | --- | --- | --- | --- | --- | --- | --- | --- | --- |
|  |  |  |  |  |  |  |  | **Lower** | **Upper** |
| **Step 1*** | **SWM total number of errors** | .099 | .046 | 4.592 | 1 | .032 | 1.104 | 1.008 | 1.208 |
| **Step 2**** | **SWM total number of errors** | .343 | .230 | 2.222 | 1 | .136 | 1.409 | .898 | 2.213 |
|  | **SOC Minimum number of moves** | -3.253 | 2.204 | 2.178 | 1 | .140 | .039 | .001 | 2.908 |

*Variables not in the equation: SWM Total number of errors, SOC Minimum number of moves, IED Total errors, IED Completed stage trials.

** Variables not in the equation: SWM Strategy, IED Total errors, IED Completed stage trials.

 Table 3. Social Cognition Cox regression model results.

|  | | **B** | **SE** | **Wald** | **df** | **Sig.** | **Exp(B)** | **95.0% CI for Exp(B)** | |
| --- | --- | --- | --- | --- | --- | --- | --- | --- | --- |
|  |  |  |  |  |  |  |  | **Lower** | **Upper** |
| **Step 1*** | **ERT Latency** | .002 | .001 | 2.181 | 1 | .140 | 1.002 | .999 | 1.005 |
| **Step 2** | **ERT Latency** | .004 | .002 | 2.046 | 1 | .153 | 1.004 | .999 | 1.008 |
|  | **ERT Correct** | -.082 | .056 | 2.188 | 1 | .139 | .0921 | .826 | 1.027 |

*Variables not in the equation: ERT Correct.

**Resting-state fMRI Results**

*Participant Data Motion Correction*

Table 4. Participant motion calculated during rs-fMRI data processing. Participants highlighted grey were excluded from subsequent fMRI analysis due to excessive motion, with a threshold of >0.15mm rmsFD implemented.

| **Participant** | **Mean rmsFD** |
| --- | --- |
| NRXN1 | 0.25 |
| NRXN2 | 0.24 |
| NRXN3 | 0.10 |
| NRXN4 | 0.14 |
| NRXN5 | 0.08 |
| NRXN6 | 0.14 |
| NRXN7 | 0.07 |
| NRXN8 | 0.09 |
| NRXN9 | 0.07 |
| NRXN10 | 0.08 |
| NRXN11 | 0.12 |
| NRXN12 | 0.07 |
| NRXN13 | 0.15 |
| NRXN14 | 0.06 |
| NRXN15 | 0.13 |
| COM1 | 0.06 |
| COM2 | 0.08 |
| COM3 | 0.08 |
| COM4 | 0.10 |
| COM5 | 0.06 |
| COM6 | 0.11 |
| COM7 | 0.11 |
| COM8 | 0.08 |
| COM9 | 0.13 |
| COM10 | 0.05 |
| COM11 | 0.15 |
| COM12 | 0.06 |
| COM13 | 0.10 |
| COM14 | 0.11 |
| COM15 | 0.19 |
| COM16 | 0.19 |

*Summary of Within-network Comparison Results*

*Visual Network Results*

Group beta = -0.075224, SE = 0.032023, 95 CI = [-0.141636, -0.008811], **p = 0.0282**

Effect size - Standardised Coefficient: -0.82; 95 CI = [-1.55, -0.10]

 Table 4. Visual network within-network comparison regression full model results.

| Coefficients | Estimate | Std Error | t | Pr(>\|t\|) |  |
| --- | --- | --- | --- | --- | --- |
| (Intercept) | 0.428276 | 0.066607 | 6.43 | 1.81E-06 | *** |
| groupNRXN | -0.075224 | 0.032023 | -2.349 | **0.0282** | * |
| mFD | -0.22082 | 0.529506 | -0.417 | 0.6807 |  |
| age | -0.002038 | 0.001243 | -1.64 | 0.1153 |  |
| gender | 0.02155 | 0.033608 | 0.641 | 0.528 |  |

***= p<0.001 ** = p< 0.01 *= p< 0.05

Residual standard error: 0.0816 on 22 degrees of freedom

Multiple R-squared: 0.3256, Adjusted R-squared: 0.203

F-statistic: 2.655 on 4 and 22 DF, p-value: 0.06014

*Somatosensory Network Results*

Group beta = -0.037678, SE = 0.042218, 95 CI = [-0.125233, 0.049878], p = 0.381812

Effect size - Standardised Coefficient: -0.31; 95 CI = [-1.03, 0.41]

 Table 5. Somatosensory network within-network comparison regression full model results.

| Coefficients: | Estimate | Std Error | t | Pr(>\|t\|) |  |
| --- | --- | --- | --- | --- | --- |
| (Intercept) | 0.433499 | 0.087811 | 4.937 | 6.14e-05 | *** |
| groupNRXN | -0.037678 | 0.042218 | -0.892 | 0.3818 |  |
| mFD | -1.847440 | 0.698077 | -2.646 | 0.0147 | * |
| age | 0.003125 | 0.001638 | 1.908 | 0.0695 |  |
| gender | 0.066630 | 0.044308 | 1.504 | 0.1468 |  |

***= p<0.001 ** = p< 0.01 *= p< 0.05

Residual standard error: 0.1076 on 22 degrees of freedom

Multiple R-squared: 0.3388, Adjusted R-squared: 0.2186

F-statistic: 2.819 on 4 and 22 DF, p-value: 0.0499

*DAN Network Results*

Group beta = -0.007871, SE = 0.032228, 95 CI = [-0.074707, 0.058965], p = 0.809311

Effect size - Standardised Coefficient: -0.10; 95 CI = [-0.97, 0.76]

 Table 6. DAN network within-network comparison regression full model results.

| Coefficients | Estimate | Std Error | t | Pr(>\|t\|) |  |
| --- | --- | --- | --- | --- | --- |
| (Intercept) | 0.2775005 | 0.0670314 | 4.140 | 0.000429 | *** |
| groupNRXN | -0.0078712 | 0.0322276 | -0.244 | 0.809311 |  |
| mFD | -0.4136549 | 0.5328816 | -0.776 | 0.445861 |  |
| age | 0.0007158 | 0.0012505 | 0.572 | 0.572836 |  |
| gender | 0.0057498 | 0.0338224 | 0.170 | 0.866563 |  |

***= p<0.001 ** = p< 0.01 *= p< 0.05

Residual standard error: 0.08212 on 22 degrees of freedom

Multiple R-squared: 0.04203, Adjusted R-squared: -0.1321

F-statistic: 0.2413 on 4 and 22 DF, p-value: 0.9118

*VAN Network Results*

Group beta = -0.060672, SE = 0.021967, 95 CI = [-0.106227, -0.015116], p = 0.011374

Effect size - Standardised Coefficient: -0.95; 95 CI = [-1.67, -0.24]

Table 7. VAN network within-network comparison regression full model results.

| Coefficients | Estimate | Std Error | t | Pr(>\|t\|) |  |
| --- | --- | --- | --- | --- | --- |
| (Intercept) | 0.3195416 | 0.0456890 | 6.994 | 5.1e-07 | *** |
| groupNRXN | -0.0606715 | 0.0219665 | -2.762 | **0.0114** | ***** |
| mFD | -0.2007465 | 0.3632153 | -0.553 | 0.5860 |  |
| age | -0.0011245 | 0.0008524 | -1.319 | 0.2006 |  |
| gender | 0.0110542 | 0.0230536 | 0.480 | 0.6363 |  |

***= p<0.001 ** = p< 0.01 *= p< 0.05

Residual standard error: 0.05597 on 22 degrees of freedom

Multiple R-squared: 0.3486, Adjusted R-squared: 0.2301

F-statistic: 2.943 on 4 and 22 DF, p-value: 0.04334

*Limbic Network Results*

Group beta = 0.014115, SE = 0.053051, 95 CI = [-0.095905, 0.124136], p = 0.792664

Effect size - Standardised Coefficient: 0.11; 95 CI = [-0.74, 0.96]

 Table 8. Limbic network within-network comparison regression full model results.

| Coefficients | Estimate | Std Error | t | Pr(>\|t\|) |  |
| --- | --- | --- | --- | --- | --- |
| (Intercept) | 0.308685 | 0.110342 | 2.798 | 0.0105 | * |
| groupNRXN | 0.014115 | 0.053051 | 0.266 | 0.7927 |  |
| mFD | 0.632557 | 0.877191 | 0.721 | 0.4784 |  |
| age | 0.001161 | 0.002058 | 0.564 | 0.5784 |  |
| gender | -0.049456 | 0.055676 | -0.888 | 0.3840 |  |

***= p<0.001 ** = p< 0.01 *= p< 0.05

Residual standard error: 0.1352 on 22 degrees of freedom

Multiple R-squared: 0.07572, Adjusted R-squared: -0.09234

F-statistic: 0.4506 on 4 and 22 DF, p-value: 0.7709

*Frontoparietal Network Results*

Group beta = -0.015007, SE = 0.029885, 95 CI = [-0.076985, 0.046971], p = 0.620549

Effect size - Standardised Coefficient: -0.19; 95 CI = [-0.99, 0.60]

 Table 9. Frontoparietal network within-network comparison regression full model results.

| Coefficients | Estimate | Std Error | t | Pr(>\|t\|) |  |
| --- | --- | --- | --- | --- | --- |
| (Intercept) | 0.141800 | 0.062159 | 2.281 | 0.0326 | * |
| groupNRXN | -0.015007 | 0.029885 | -0.502 | 0.6205 |  |
| mFD | -0.430168 | 0.494149 | -0.871 | 0.3934 |  |
| age | 0.001748 | 0.001160 | 1.507 | 0.1460 |  |
| gender | 0.059023 | 0.031364 | 1.882 | 0.0731 |  |

***= p<0.001 ** = p< 0.01 *= p< 0.05

Residual standard error: 0.07615 on 22 degrees of freedom

Multiple R-squared: 0.1913, Adjusted R-squared: 0.04427

F-statistic: 1.301 on 4 and 22 DF, p-value: 0.3003

*Default Network Results*

Group beta = -0.015803, SE = 0.024260, 95 CI = [-0.066115, 0.034510], p = 0.521543

Effect size - Standardised Coefficient: -0.26; 95 CI = [-1.10, 0.57]

 Table 10. Default Mode network within-network comparison regression full model results.

| Coefficients | Estimate | Std Error | t | Pr(>\|t\|) |  |
| --- | --- | --- | --- | --- | --- |
| (Intercept) | 0.2340532 | 0.0504597 | 4.638 | 0.000127 | *** |
| groupNRXN | -0.0158028 | 0.0242602 | -0.651 | 0.521543 |  |
| mFD | -0.5722845 | 0.4011415 | -1.427 | 0.167722 |  |
| age | 0.0003279 | 0.0009414 | 0.348 | 0.730913 |  |
| gender | 0.0090658 | 0.0254608 | 0.356 | 0.725180 |  |

***= p<0.001 ** = p< 0.01 *= p< 0.05

Residual standard error: 0.06182 on 22 degrees of freedom

Multiple R-squared: 0.1129, Adjusted R-squared: -0.04845

F-statistic: 0.6996 on 4 and 22 DF, p-value: 0.6004

*Subcortical Network Results*

Group beta = 0.009163, SE = 0.021503, 95 CI = [-0.035432, 0.053758], p = 0.674155

Effect size - Standardised Coefficient: 0.15; 95 CI = [-0.60, 0.91]

Table 11. Subcortical network within-network comparison regression full model results.

| Coefficients | Estimate | Std Error | t | Pr(>\|t\|) |  |
| --- | --- | --- | --- | --- | --- |
| (Intercept) | 0.2216722 | 0.0447252 | 4.956 | 5.86e-05 | *** |
| groupNRXN | 0.0091631 | 0.0215032 | 0.426 | 0.6742 |  |
| mFD | -0.8069405 | 0.3555534 | -2.270 | 0.0334 | * |
| age | 0.0016170 | 0.0008344 | 1.938 | 0.0656 |  |
| gender | 0.0126279 | 0.0225673 | 0.560 | 0.5814 |  |

***= p<0.001 ** = p< 0.01 *= p< 0.05

Residual standard error: 0.05479 on 22 degrees of freedom

Multiple R-squared: 0.2766, Adjusted R-squared: 0.1451

F-statistic: 2.103 on 4 and 22 DF, p-value: 0.1148

*Summary of Network Based Statistic Results*

Table 12. Network Based Statistic ROI labels, network assignments and centroid coordinates (x,y,z), for 100 Scahefer ROIs and 15 subcortical ROIS.

| **ROI Name and Network** | **Coordinates** | | |
| --- | --- | --- | --- |
| **100 Schaefer ROIs** | **x** | **y** | **z** |
| Left Visual ROI1 | -25 | -32 | -17 |
| Left Visual ROI2 | -25 | -76 | -13 |
| Left Visual ROI3 | -16 | -61 | -6 |
| Left Visual ROI4 | -27 | -94 | -4 |
| Left Visual ROI5 | -5 | -92 | -2 |
| Left Visual ROI6 | -12 | -65 | 6 |
| Left Visual ROI7 | -47 | -70 | 10 |
| Left Visual ROI8 | -25 | -88 | 20 |
| Left Visual ROI9 | -7 | -81 | 25 |
| Left Somatomotor ROI1 | -53 | -22 | 7 |
| Left Somatomotor ROI2 | -37 | -21 | 15 |
| Left Somatomotor ROI3 | -54 | -11 | 13 |
| Left Somatomotor ROI4 | -54 | -8 | 33 |
| Left Somatomotor ROI5 | -39 | -23 | 58 |
| Left Somatomotor ROI6 | -10 | -26 | 63 |
| Left Dorsal Attention Network (DAN) (Posterior) ROI1 | -46 | -56 | -12 |
| Left Dorsal Attention Network (DAN) (Posterior) ROI2 | -57 | -25 | 38 |
| Left Dorsal Attention Network (DAN) (Posterior) ROI3 | -25 | -68 | 48 |
| Left Dorsal Attention Network (DAN) (Posterior) ROI4 | -41 | -34 | 48 |
| Left Dorsal Attention Network (DAN) (Posterior) ROI5 | -5 | -59 | 56 |
| Left Dorsal Attention Network (DAN) (Posterior) ROI6 | -22 | -50 | 66 |
| Left Dorsal Attention Network (DAN) (Frontal) ROI1 | -48 | 6 | 27 |
| Left Dorsal Attention Network (DAN) (Frontal) ROI2 | -27 | -2 | 58 |
| Left Ventral Attention Network (VAN) (Frontal) ROI1 | -58 | -37 | 30 |
| Left Ventral Attention Network (VAN) (Anterior) ROI1 | -41 | -1 | -6 |
| Left Ventral Attention Network (VAN) (Anterior) ROI2 | -38 | 12 | 6 |
| Left Ventral Attention Network (VAN) (Lateral) ROI1 | -29 | 43 | 29 |
| Left Ventral Attention Network (VAN) (Medial) ROI1 | -5 | 19 | 33 |
| Left Ventral Attention Network (VAN) (Medial) ROI2 | -10 | -34 | 45 |
| Left Ventral Attention Network (VAN) (Medial) ROI3 | -6 | 3 | 61 |
| Left Limbic (Orbital) ROI1 | -13 | 32 | -19 |
| Left Limbic (Temporal) ROI1 | -33 | 1 | -34 |
| Left Limbic (Temporal) ROI2 | -56 | -32 | -21 |
| Left Frontoparietal (Posterior) ROI1 | -37 | -54 | 46 |
| Left Frontoparietal (lateral Prefrontal Cortex) ROI1 | -43 | 32 | 20 |
| Left Frontoparietal (Posterior Medial) ROI1 | -9 | -73 | 37 |
| Left Frontoparietal (Medial) ROI1 | -3 | -26 | 32 |
| Left Default (Temporal) ROI1 | -55 | -3 | -20 |
| Left Default (Temporal) ROI2 | -57 | -31 | -1 |
| Left Default (Temporal) ROI3 | -57 | -50 | 11 |
| Left Default (Temporal) ROI4 | -47 | -63 | 35 |
| Left Default (Frontal) ROI1 | -34 | 21 | -11 |
| Left Default (Frontal) ROI2 | -46 | 33 | -1 |
| Left Default (Frontal) ROI3 | -5 | 46 | 0 |
| Left Default (Frontal) ROI4 | -23 | 59 | -1 |
| Left Default (Frontal) ROI5 | -9 | 44 | 39 |
| Left Default (Frontal) ROI6 | -41 | 14 | 47 |
| Left Default (Frontal) ROI7 | -25 | 20 | 51 |
| Left Default (Posterior Cingulate) ROI1 | -11 | -55 | 13 |
| Left Default (Posterior Cingulate) ROI2 | -5 | -53 | 32 |
| Right Visual ROI1 | 31 | -30 | -21 |
| Right Visual ROI2 | 27 | -65 | -11 |
| Right Visual ROI3 | 49 | -60 | -11 |
| Right Visual ROI4 | 22 | -93 | -3 |
| Right Visual ROI5 | 8 | -76 | 4 |
| Right Visual ROI6 | 16 | -57 | 6 |
| Right Visual ROI7 | 36 | -81 | 16 |
| Right Visual ROI8 | 13 | -86 | 28 |
| Right Somatomotor ROI1 | 53 | -15 | 6 |
| Right Somatomotor ROI2 | 40 | -15 | 14 |
| Right Somatomotor ROI3 | 56 | -4 | 11 |
| Right Somatomotor ROI4 | 57 | -5 | 30 |
| Right Somatomotor ROI5 | 46 | -10 | 48 |
| Right Somatomotor ROI6 | 41 | -21 | 59 |
| Right Somatomotor ROI7 | 30 | -37 | 63 |
| Right Somatomotor ROI8 | 11 | -24 | 65 |
| Right Dorsal Attention Network (DAN) (Posterior) ROI1 | 50 | -62 | 15 |
| Right Dorsal Attention Network (DAN) (Posterior) ROI2 | 49 | -23 | 42 |
| Right Dorsal Attention Network (DAN) (Posterior) ROI3 | 39 | -45 | 49 |
| Right Dorsal Attention Network (DAN) (Posterior) ROI4 | 26 | -66 | 50 |
| Right Dorsal Attention Network (DAN) (Posterior) ROI5 | 14 | -52 | 65 |
| Right Dorsal Attention Network (DAN) (Frontal) ROI1 | 48 | 9 | 27 |
| Right Dorsal Attention Network (DAN) (Frontal) ROI2 | 28 | -2 | 58 |
| Right Ventral Attention Network (VAN) (Lateral) ROI1 | 58 | -42 | 13 |
| Right Ventral Attention Network (VAN) (Lateral) ROI2 | 60 | -25 | 27 |
| Right Ventral Attention Network (VAN) (Anterior) ROI1 | 40 | 8 | 0 |
| Right Ventral Attention Network (VAN) (Medial) ROI1 | 10 | -30 | 45 |
| Right Ventral Attention Network (VAN) (Medial) ROI2 | 7 | 5 | 52 |
| Right Limbic (Orbital) ROI1 | 12 | 36 | -19 |
| Right Limbic (Temporal) ROI1 | 38 | 0 | -35 |
| Right Frontoparietal (Posterior) ROI1 | 57 | -38 | 43 |
| Right Frontoparietal (Posterior) ROI2 | 45 | -62 | 45 |
| Right Frontoparietal (lateral Prefrontal Cortex) ROI1 | 29 | 57 | -2 |
| Right Frontoparietal (lateral Prefrontal Cortex) ROI2 | 45 | 38 | 15 |
| Right Frontoparietal (lateral Prefrontal Cortex) ROI3 | 31 | 45 | 29 |
| Right Frontoparietal (lateral Prefrontal Cortex) ROI4 | 43 | 16 | 45 |
| Right Frontoparietal (medial Prefrontal Cortex) ROI1 | 5 | -27 | 32 |
| Right Frontoparietal (medial Prefrontal Cortex) ROI2 | 6 | 26 | 31 |
| Right Frontoparietal (medial Prefrontal Cortex) ROI3 | 10 | -64 | 42 |
| Right Default Parietal ROI1 | 54 | -50 | 30 |
| Right Default (Temporal) ROI1 | 61 | -23 | -17 |
| Right Default (Temporal) ROI2 | 50 | 6 | -17 |
| Right Default (Temporal) ROI3 | 57 | -26 | -2 |
| Right Default ventral Prefrontal Cortex ROI1 | 35 | 26 | -13 |
| Right Default ventral Prefrontal Cortex ROI2 | 50 | 28 | 0 |
| Right Default medial Prefrontal Cortex ROI1 | 6 | 48 | 0 |
| Right Default medial Prefrontal Cortex ROI2 | 11 | 45 | 39 |
| Right Default medial Prefrontal Cortex ROI3 | 26 | 24 | 49 |
| Right Default (Posterior Cingulate) ROI1 | 12 | -54 | 14 |
| Right Default (Posterior Cingulate) ROI2 | 7 | -52 | 30 |
| **15 Subcortical ROIs** |  |  |  |
| Subcortical ROI1 - Left Thalamus | -9 | -19 | 6 |
| Subcortical ROI2 - Left Caudate | -12 | 9 | 9 |
| Subcortical ROI3 - Left Putamen | -24 | 0 | 0 |
| Subcortical ROI4 - Left Pallidum | -19 | -4 | -1 |
| Subcortical ROI5 - Brainstem | 0 | -31 | -34 |
| Subcortical ROI6 - Left Hippocampus | -24 | -22 | -14 |
| Subcortical ROI7 - Left Amygdala | -22 | -5 | -18 |
| Subcortical ROI8 - Left Nucleus Accumbens | -9 | 11 | -7 |
| Subcortical ROI9 - Right Thalamus | 10 | -18 | 6 |
| Subcortical ROI10 - Right Caudate | 13 | 10 | 10 |
| Subcortical ROI11 - Right Putamen | 25 | 1 | 0 |
| Subcortical ROI12 - Right Pallidum | 20 | -3 | -1 |
| Subcortical ROI13 - Right Hippocampus | 26 | -20 | -14 |
| Subcortical ROI14 - Right Amygdala | 22 | -3 | -17 |
| Subcortical ROI15 - Right Nucleus Accumbens | 9 | 12 | -6 |
